# Primary prevention programs for childhood obesity: are they cost-effective?

**DOI:** 10.1186/s13052-023-01424-9

**Published:** 2023-03-02

**Authors:** Miriana Guarino, Lorena Matonti, Francesco Chiarelli, Annalisa Blasetti

**Affiliations:** University of Study G. d’Annunzio, Chieti-Pescara, Italy

**Keywords:** Cost-effectiveness, Childhood overweight and obesity, Adolescence overweight and obesity, Primary prevention

## Abstract

Childhood obesity is increasing all over the world. It is associated with a reduction in quality of life and a relevant burden on society costs. This systematic review deals with the cost-effectiveness analysis (CEA) of primary prevention programs on childhood overweight/obesity, in order to benefit from cost-effective interventions.

We screened and evaluated all the studies with a cost-effectiveness analysis on childhood obesity primary prevention program by PUBMED and Google Scholar, using inclusion and exclusion criteria. The quality of the studies was assessed by Drummond’s checklist.

Ten studies were included. Two of them examined the cost-effectiveness of community-based prevention programs, four focused only on school-based programs while four more studies examined both community-based and school-based programs. The studies were different in terms of study design, target population, health and economic outcomes. Seventy per cent of the works had positive economic results.

The majority of the studies showed effective economic outcomes applying primary prevention programs on childhood obesity. It is important to increase homogeneity and consistency among different studies.

## Introduction

Overweight and obesity are due to abnormal and excessive fat accumulation caused by an energy imbalance between calories consumed and calories expended, that may impair health. [1] The definition of obesity differs according to sex and age: in children up to 24 months, the diagnosis of obesity is based on weight-for-length, while in children from 2 to 5 years old and from 5 to 19 years old, obesity is defined as body mass index (BMI) > 3 SD and > 2 SD above the mean of the WHO international growth standards respectively. [1, 2] Based on recent data, overweight and obese children are 41 million and it is predicted to increase to 70 million globally by 2025. [3]

The consequences of overweight and obesity are already evident in childhood with long-term consequences up to adulthood, indeed they can be affected by gastrointestinal, musculoskeletal and orthopedic diseases, behavioral and emotional problems. Moreover, they have an increased risk of early onset of insulin resistance and type 2 diabetes [4], along with obesity, cardiovascular disease, cancers, premature death and disability in adulthood. [5, 6] In particular, obese children have cardio-metabolic changes early in life, with a major risk of cardiovascular disease in adulthood and a consequent increased risk of premature morbidity and mortality [7]. Numerous publications show the childhood origin of cardiovascular disease; in particular elevated blood pressure levels in children play a role in the development of increasing left ventricular mass (LVM) in both children and adults and then concentric left ventricular hypertrophy (LVH) [8].

Overweight and obesity entail also direct and indirect economic consequences. [9] The direct costs relate to the healthcare needs, while the indirect costs are a result of productivity losses (disability and premature death) [10].

Primary prevention is possible by lifestyle changes, [11] in particular in the first 1000 days [12] and in the preschool years [13]. The first 1000 days, the period between conception (day 18) and the age of two years, are considered a window of opportunity for obesity prevention. Nutritional environment during prenatal and neonatal period may interfere with genome regulation, resulting in permanent changes in body structure and function. This adaptative response could lead to chronic diseases like metabolic syndrome, cardiovascular diseases, hypertension, and obesity [14].

During the first 1000 days, several modifiable risk factors are associated to later childhood obesity, such as pre-conception factors (e.g. higher maternal pre-pregnancy BMI), post conception factors (e.g. excessive gestational weight gain and high infant birth weight) and other factors (e.g. parent-infant relationship) [15].

Early-life intervention based on these modifiable factors is critical for pediatric and adult obesity prevention. [16] Multi-component interventions targeting diet and physical activity in preschool children have shown some success in reducing obesity risk [17], such as removing TVs from bedrooms, providing alternative playtime activities, implementing the intake of water, low-fat milk, fruit and vegetables as snacks and increasing hours of physical activity (PA) [18, 19].

Cost-effectiveness analysis is the method that compares various interventions in terms of their costs in order to discover the cheapest and most efficient intervention program about people’s well-being; this analysis may point out the need for evidence-based policy making for obesity prevention [18–20]. Economic evaluation is a mean to optimize society’s welfare. In many countries, the scarcity of economic resources relative to needs requires decision makers to prioritize their use in a correct way [18–20]. Cost-effectiveness analysis is based on the economic evaluation of anthropometric measurements in the population, like BMI and waist circumference, and the evaluation of primary outcomes, like disability-adjusted life years (DALYs), quality-adjusted life years (QALYs), kilograms (kg) weight loss, % body fat, BMI (z-score), in the economic setting of cost-saving. In particular, the DALYs saved owing to a prevention intervention is considered the difference in future mortality and morbidity outcomes between a baseline scenario (base case), which represents current practice, and the intervention option, while QALYs is a measure that combines lifespan with its quality (the value of 1 indicates the life expectancy of one year in good health) [18–20]. Lastly, the incremental cost-effectiveness ratio (ICER) is a statistic index used to synthesized the cost-effectiveness of a health care intervention. It represents the average incremental cost associated with one additional unit of the measure of effect [18–20].

The purpose of our systematic review is to assess the outcomes reported within previous economic evaluations from studies in the literature and to determine whether it is economically viable to apply obesity primary prevention programs. In particular, our work evaluates the cost-effectiveness of prevention programs.

## Materials and methods

### Eligibility criteria and search

We performed a systematic review considering all published studies in English from January 2000 till 31^st^ December 2021, in which a cost-effectiveness analysis of childhood obesity primary prevention program appears. This review was written according to Preferred Reporting Items for Systematic reviews and Meta-Analysis (PRISMA) statement. Inclusion and exclusion criteria were clearly defined.

Inclusion criteria were the following:

- 1. Randomized clinical trials (RCTs);
- 2. Systematic reviews;
- 3. Cohort studies;
- 4. Model based studies;
- 5. Children and adolescence of preschool and school age;
- 6. Community-based and school-based intervention programs;
- 7. Presence of health and economic outcomes.

Short notes, study protocols, abstracts, editorials and studies without an economic evaluation outcome were excluded.

We conducted the search by PUBMED and Google Scholar with the following key words: “cost-effectiveness” AND “childhood overweight and obesity” AND “adolescence overweight and obesity” AND “primary prevention”.

### Measures

There are many health outcome measures, in particular QALYs (Quality-Adjusted Life Years), DALYs (Disability-Adjusted Life Years), HALYs (Health-adjusted life years), BMI, BMI z-scores and increased unit BMI avoided, kilograms (kg) weight gain prevented and percentage of body fat reduction, number of obesity cases avoided, reduction of obesity prevalence, life-years gained and weight-related event avoided, waist circumference and unit (0.01) waist-to-height ratio (WHtR) increase prevented. These measures give the cost of the primary prevention programs designed.

Economic measures adopted were Cost-Effectiveness (CER) and Incremental Cost-Effectiveness (ICER) ratios per DALYs, HALYs, QALYs, increased unit BMI avoided, reduction unit of BMI z-score, waist circumference (WC) and unit (0.01) waist-to-height ratio (WHtR) increase prevented, number of obesity cases avoided, reduction of obesity prevalence, cost of intervention per kg weight gain prevented or percentage of body fat reduction, cost per life-year gained and cost per weight-related event avoided, healthcare-related cost offsets from diseases, cost per BMI unit change and net costs.

### Quality assessment

We assessed the articles for eligibility using the Drummond’s checklist, that evaluates the economic aspects and it considers: 1) the research question; 2) the description of the study/intervention; 3) the study design; 4) the identification, measurement and evaluation of costs and consequences; 5) whether discounting was carried out; 6) incremental analysis; 7) presentation of results with uncertainty and sensitivity analyses; and 9) discussion of results [21, 22].

We analyzed the articles one by one, in order to answer questions from the checklist. We put “YES” if the article clearly expressed a positive answer to the question; we put “NOT” if the article did not have a specific characteristic, while we put “NOT CLEAR” if the article did not highlight a specific aspect. Each author had a defined number of studies to analyze.

## Results

We searched articles by keywords through PUBMED and Google Scholar. The search produced a total of 63 articles through PUBMED and 520 articles through Google Scholar; 40 studies were in common between the two searches.

**Fig. 1:**
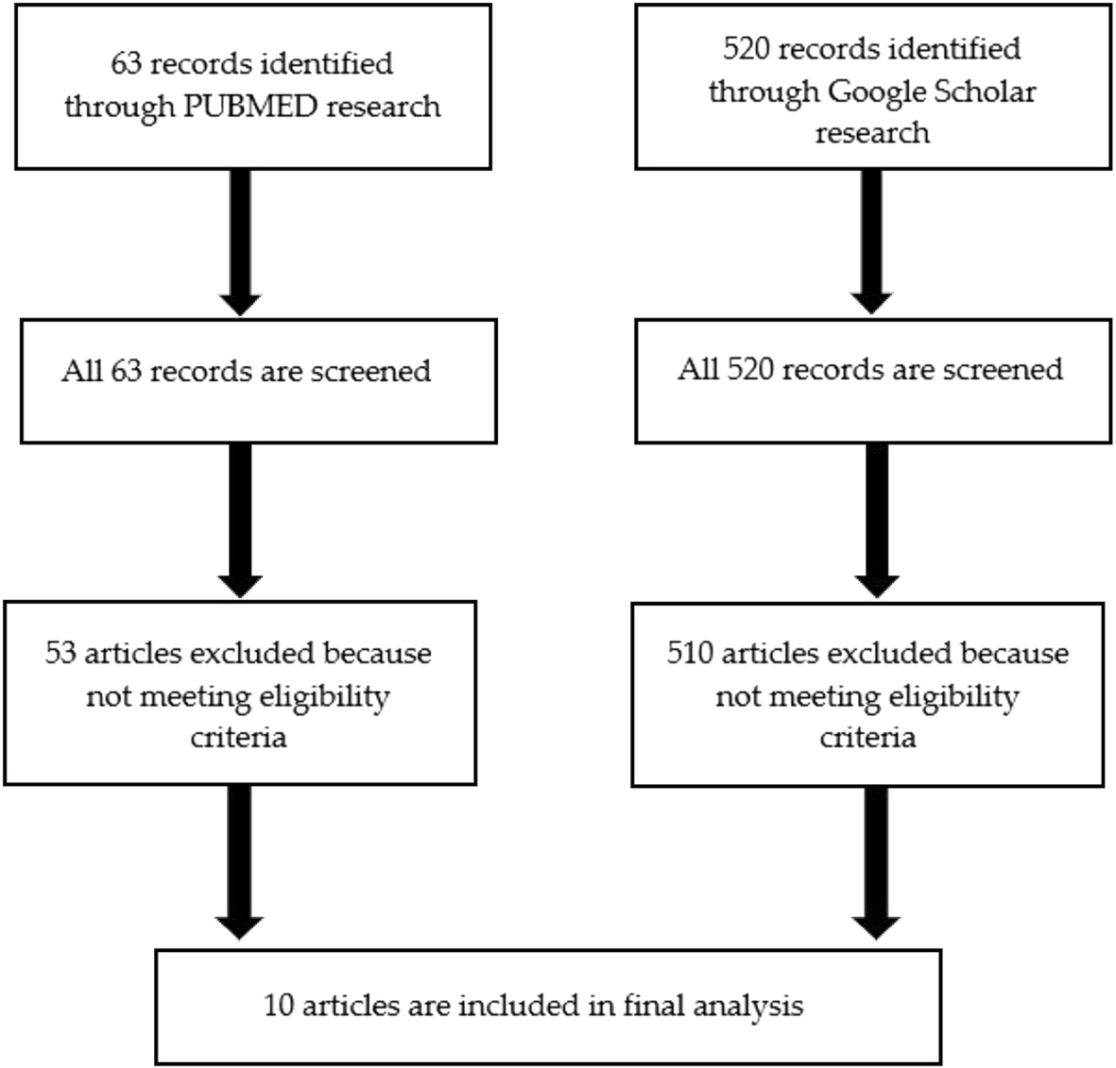
Flow of information for systematic review

We excluded, respectively, 53 and 510 articles because they did not meet inclusion criteria, so 10 studies were eligible for the review. Many of them used QALY like health outcomes, [23–27] less DALY, HALY, changes in BMI and weight and life-year gained. [23, 25–32] Fig. 1 represents the screening and eligibility of the articles.

## Studies description

All the studies analyzed the cost-effectiveness of childhood obesity primary prevention programs. Two of them examined cost-effectiveness of community-based intervention programs, four were only school-based programs while four of them examined both community-based and school-based intervention programs. The studies were different in study design, characteristics of population and in health and economic outcomes. In addition, there was heterogeneity about the type of intervention program used. Some articles did not clarify the childhood age range evaluated.

An intervention study considered the economic evaluation of the URMEL-ICE (Ulm Research on Metabolism, Exercise and Lifestyle Intervention in Children), an overweight school-based prevention program, based on developed teaching materials including health education (consumption of sweetened beverages and media use), physical activity and parent involvement in the primary schools. The basic study was a school-based, cluster-randomized intervention trial conducted in the region of Ulm and Günzburg, in Germany [31].

Differences in BMI, WC and WHtR were calculated. Intervention costs were €24.09 per child. The maximum willingness to pay (MWTP) was €35. The ICER (Incremental cost-effectiveness ratio) for WC and WHtR was calculated. ICER was €11.11 per cm WC growth inhibited and €18.55 per unit (0.01) WHtR increase avoided, so the study showed favourable cost-effectiveness ratios. The results proved that prevention of overweight and obesity in a school setting could be cost-effective.

The systematic review of Erdöl Ş et al. included eight studies, five of which concerning community-based primary intervention programs, while three of them were school-based programs. [23] Health outcome measures were evaluated by DALYs, QALYs, BMI scores, kilograms (kg) weight gain prevented and percentage of body fat reduction. The economic measures consisted in Cost Effectiveness (CER) and Incremental Cost Effectiveness ratios for DALY and QALY and cost of intervention per kg weight gain prevented. In summary, three school-based and one community-based primary prevention programs reported were cost effective: two of them considered cost per QALY, one cost for DALY and the last cost per percentage point body fat reduction, while the other studies were not cost effective. Thus, fifty-percent of the studies had positive results. In addition, we have to consider that these studies are low-medium quality and heterogeneous in study designs and outcome measures.

The “Healthy Beginnings” (HB) was an early childhood community-based program, a randomized clinical trial, delivered to families in socio-economically disadvantaged areas of Sydney, Australia during 2007–2010. [28] The HB program consisted of eight home visits by specially trained community nurses, from the 30–36 weeks gestational age up to age two years, with age-appropriate education and advice on feeding, nutrition and physical activity. Health measures were BMI and BMI z-score. Net costs evaluated direct and healthcare costs, in particular the cost of HB intervention in the clinical trial over two years was $1309 per child. Cost-effectiveness analysis was estimated by ICER per BMI unit avoided and cost per 0.1 BMI z-score reduction. The incremental cost-effectiveness ratio was $4230 per unit BMI avoided and $631 per 0.1 reduction in BMI z-score. If the trial reduced the travel time for home visits, it was estimated that the program could costs $709 per child; with incremental cost-effectiveness ratios of $2697 per unit BMI avoided and $376 per 0.1 reduction in BMI z-score. The analyses showed a BMI reduction among the first 2 years of life. The economic analyses showed only short-term benefits of the program using 2 years outcome data, without benefits on longer term. Thus, HB was a moderately priced intervention, with an increased cost-effectiveness if the travel time for home visits was reduced.

A recent overview paper reported the initial results from the Childhood Obesity Intervention Cost-Effectiveness Study (CHOICES), a model to estimate cost-effectiveness of interventions to reduce childhood obesity in the U.S. [25] There were four initial interventions, both community-based and school-based, like childhood obesity intervention programs.

The interventions were the following:

- an excise tax of $0.01 per ounce sugar-sweetened beverages (SSB);
- elimination of the tax deductibility of advertising costs of TV advertisements for “nutrient-poor” food and beverages seen by children and adolescents (TV AD);
- physical education in public elementary school ≥ 50% of physical education class time to moderate and vigorous physical activity (Active PE);
- early child educational setting by increasing physical activity, improving nutrition and reducing screen time (ECE).

The outcomes evaluated in shorter-term (2 years) and longer-term (10 years) were cost per BMI unit change for 2 years following an intervention and 10-year healthcare cost, net cost, DALYs and QALYs. The estimated cost-effectiveness of the interventions for the first two years varied more, ranging from a low of $1.16 per BMI unit change for TV AD, to $3.16 for SSB, $ 57.80 for ECE and $401 for the Active PE intervention. Substantial variations were in 10-year health outcomes (net cost saved per $ spent: $55 for SSB, $38 for TV AD, $6 for ECE). The major saving was for SSB intervention ($23.2 billion) because this impacted on all age groups. At last, SSB intervention averted DALYs and both SSB and TV AD increased QALYs. These results had a lower cost than some medical treatments (e.g. bariatric surgical intervention).

However, this review examined studies with high heterogeneity, in which the population was different in age and with an undefined starting BMI.

Similar to the previous study, other authors developed a microsimulation model to evaluate the cost-effectiveness of seven different interventions identified as potentially important strategies for addressing childhood obesity (both community-based and school-based). [32] The interventions were: an excise tax of one cent per ounce on sugar-sweetened beverages, applied nationally; the elimination of the tax deductibility of advertising costs for television ads seen by children for nutrient-poor food and beverages; restaurant menu calorie labeling; implementation of nutrition standards for federally reimbursable school meals sold; implementation of nutrition standards for all food and beverages sold in schools outside of reimbursable school meals; improved early childhood education policies and practices and a nationwide fourfold increase in the use of adolescent bariatric surgery. The study calculated costs per BMI units reduced over two years (2015–17), health-care costs, net costs, and net costs saved per dollar spent over ten years (2015–25). Moreover, it was estimated the number of obesity cases prevented and changes in childhood obesity prevalence in 2025. In brief, eliminating the tax deduction for advertising nutrient-poor food reduced a BMI unit for $0.66 per person, while increasing access to bariatric surgery could reduce a BMI unit for $1,611. Three of these interventions were projected to save more and reduce health costs (the sugar-sweetened beverage excise tax, eliminating the tax subsidy for advertising unhealthy food to children and setting nutrition standards for food and beverages sold in schools outside of school meals). For example, the beverage excise tax would save $14.2 billion in net costs, over the decade 2015–2025, primarily due to reductions in adult health care costs. Important was the prevention of 576,000, 129,100, and 345,000 cases of childhood obesity, respectively, in 2025, while the net savings to society for each dollar spent were projected to be $30.78, $32.53, and $4.56, respectively.

Conesa and colleagues carried out a cost-effectiveness analysis of a primary prevention school-based intervention of childhood obesity, the EdAI (Educació en Alimentació). [29] It was a randomized, controlled primary prevention intervention implemented in various school of Catalonia, Spain, by healthy lifestyle choices through diet and physical activity (increasing fruit, vegetables, legumes and fish intake for children aged 7–8 years over a period of 28 months). The study analyzed changes in BMI z-score and obesity prevalence and ICER for the number of obesity cases avoided, the decrease in obesity prevalence, the decrease in BMI units, and the decrease in BMI z-score units from the beginning to the end of the intervention was calculated. The ICER was performed only for boys because the intervention was not effective for obesity-related outcomes for girls. After 28 months, the obesity prevalence decreased significantly (by 2.02%) in the intervention group and increased by 0.44% in the control group. In particular for boys, the intervention group exhibited an effective reduction of − 0.24 units in the BMI z-score compared with the control group. Total cost of intervention was 15.64 € per child or 5.21 €/child/year. About ICER, 968.66 € to avoid one case of obesity in boys (1.20 € per boy); 3.56 €/child to reduce the obesity prevalence by 1% in boys; 47.39 € for a decrease of one BMI unit per boy; and 65.17 € for a decrease of one BMI z-score unit per boy. In conclusion, despite gender differences in outcome, EdAI costs 2.4 € per child per year to achieve a greater than 2% reduction in the obesity prevalence, as requested by the 2009 cost effectiveness criteria of the Spanish Health Ministry.

The HELP program was a school-based obesity prevention program in children aged 9–10 years, through primary schools in south-west England, with a follow-up at 24 months. [27] The program consisted of four steps: (1) building a receptive environment, (2) a drama-based healthy lifestyles week, (3) one-to-one goal setting, (4) reinforcement activities. The health outcomes were BMI standard deviation score (SDS) at 24 months post the beginning of the intervention, waist circumference SDS, percentage body fat SDS, proportion of children overweight at 18 and 24 months, accelerometer-assessed physical activity and food intake at 18 months. The study reported no difference in BMI SDS at 24 months or at 18 months, and no differences in waist circumference SDS, percentage body fat SDS or physical activity levels between the intervention and control groups. At 18 months, children in the intervention group consumed less energy-dense snacks compared to children in the control group. The cost of the HELP program was approximately £144,749, with a mean estimated cost per child of £214. The study showed that there were not improvements in the incidence of the weight-related health events or cost savings to the health and social care system associated with weight-related events. The study estimated cost-effectiveness analyses, cost-per-life-year and cost-per-QALY. Through this model, the cost-effectiveness of public health interventions typically resulted in the use of a long-term time horizon, often over 30–40 years, the mean incremental differences in costs and outcomes were relatively small. This is the first no cost-effective study in preventing overweight or obesity in children aged 9–10 years.

In 2019 Canaway published the economic analysis of “WAVES” (The West Midlands ActiVe lifestyle and healthy Eating in School children), a childhood obesity school-based preventing program. [24] The 12-month intervention, in children aged 6–7 years from 54 schools across the West Midlands (UK), was based on the increasing physical activity by 30 min per day and encourage healthy eating. QALYs was calculated as health outcome by using the CHU-9D quality of life questionnaire, validated for use in a pediatric population. The mean difference in QALYs between the group of children who received the intervention and the control group at follow up was 0.006. The incremental cost of the intervention compared to the control arm was £155.53 per child. The incremental cost-effectiveness at 30 months was £26,815 per QALY, less than the standard willingness to pay threshold of £30,000 per QALY, but the calculated probability of the WAVES intervention being cost-effective was only 52%. Therefore, it was not clear if the WAVES program was a cost-effective obesity childhood preventing intervention, because there was a small incremental QALY gain and a major cost when compared to the control group, so it is likely that the incremental of QALYs was due to underlying baseline differences between intervention and control arm and not to an effective health gain.

An Australian study examined the cost-effectiveness of community-based and school-based obesity prevention interventions (CBIs), defined as a community-based program to promote healthy eating and physical activity for children aged 5–18 years. [30] The CBIs consisted in capacity building, awareness raising, physical activity and nutrition strategies implemented in schools, infrastructure changes to schools, and changes to food and physical activity environments within the community. The study was a cost-effectiveness analysis of a review and meta-analysis. The change in BMI has been evaluated for individual age and sex groups from 5–18 years and the health-adjusted life years (HALYs). The meta-analysis revealed a difference in BMI z-score promoting the CBI community compared with the control community in children aged 5–18 years. The estimated net cost of implementing CBIs across all local government areas in Australia was AUD426M over three years. Incremental cost-effectiveness ratio was calculated. If ICER was less than the commonly used willingness to pay threshold for Australia of AUD 50,000 per HALY gained, the intervention was considered cost-effective. The ICER had a value of AUD8155 per HALY gained. CBIs was cost-effective obesity prevention initiatives; however, implementation across Australia would be relatively expensive when compared with current investments in preventive health. Thus, the interventions would be cost-effective over a 29-year time horizon.

Another early community-based intervention program analysis evaluated if childhood overweight prevention intervention, like sleep intervention alone or with food, activity, and breastfeeding advice was cost-effective compared with usual care. [26] The cost-effectiveness analysis was based on Prevention of Overweight in Infancy (POI) clinical trial, that showed an important reduction risk of obesity through sleep intervention, with or without food, activity, and breastfeeding advice (FAB), within the first two years of life. The POI trial included a 3.5- and 5-years follow-up. The Sleep intervention consisted of contact by trained research staff, in which participants received advice about how to promote healthy infant sleep: identifying when infants were tired, helping infants learn how to settle themselves to sleep, and not using feeding as the first response to infant distress. The FAB intervention consisted of parent contact with information on nutrition, physical activity, and breastfeeding. The cost-effectiveness analysis began at five years and modeled cost-effectiveness and cost-utility analysis to age 15 years for both Sleep and Combination interventions, to evaluate the period between early childhood and adolescence. The health outcomes were BMI at age 5 years and QALYs at age 15 years. QALYs was based only on child weight status and the net mean difference in effectiveness was 0.008 in the Sleep group and 0.006 in the combination group for QALYs. The ICERs was calculated as incremental cost per QALY gained and incremental cost per unit BMI avoided in the intervention compared with control. The average costs of the Sleep and Combination interventions were $184 and $601 per child, respectively, with ICER for the Sleep intervention of $18,125 per QALY gained and the ICER for the Combination intervention of $94,667 per QALY gained, while the ICER was $1,022 per unit BMI avoided at age 5 years and $558 per unit BMI avoided at age 15 years in the Sleep group. ICER for combination intervention was higher ($6,678 and $5,164 respectively). Thus, we had 74% probability for Sleep intervention of being cost-effective at a willingness-to-pay threshold of $50,000 per QALY, while the Combination intervention had a 23% probability of being cost-effective. In fact, the addition of FAB in the Combination intervention greatly increased the cost per child with an ICER not cost-effective. In addition, the cost-effectiveness analyses measured in BMI avoided showed that the Sleep intervention was more cost-effective than the Combination program at all willingness-to-pay thresholds. The study concluded that only the Sleep intervention, without food, activity and breastfeeding advice, was a cost-effective approach to prevent childhood obesity, while both interventions were cost-effective over a 15-year time horizon because there was some money saving in health care costs and incremental BMI that occurs during adolescence.

Table 1 summarizes the characteristics of studies included in the systematic review.

**Table 1:** Description of characteristics of the studies included in the systematic review

Study	Type of intervention	Health outcome	Cea result
**Kesztyu’s 2011** [31]	School-based program	BMI, WC, WHtR	Cost-effective
**Erdol 2014** [23]	Community-based and school-based programs	DALYs, QALYs, BMI z-score, kg weight gain prevented, % of body fat reduction	50% of the studies are cost-effective 50% of the studies are not cost-effective
**Hayes 2014** [28]	Community-based program	BMI, BMI z-score	Moderately cost-effective
**Gortmaker 2015 (CHOICE)** [25]	Both community-based and school-based programs	BMI unit change, DALYs, QALYs	Cost-effective
**Gortmaker 2015 (Obesity & Diet)** [32]	Community-based and school-based programs	BMI unit change, healthcare cost	Cost-effective
**Conesa 2018** [29]	School-based program	BMI z-score, obesity prevalence	Cost-effective
**Wyatt 2018** [27]	School-based program	BMI-WC-% body fat SDS QALYs	Not cost-effective
**Canaway 2019** [24]	School-based program	QALYs	Not clear cost-effective
**Ananthap. 2019** [30]	Both community-based and school-based programs	BMI changes, HALYs	Cost-effective
**Joo Tan 2020** [26]	Community-based program	BMI, QALYs	Cost-effective

Table 2 summarizes economic outcomes.

**Table 2:** Description of economic outcomes

Study	Economic outcomes
**Kesztyu’s 2011** [31]	**INTERVENTION COSTS:** €24.09 per child **ICER:** €11.11 per cm WC growth inhibited and €18.55 per unit (0.01) WHtR increase avoided
**Erdol 2014** [23]	**ICER:** Four of the eight studies have positive economic outcomes (two of them considered cost per QALY, one cost for DALY and one cost per % point body fat reduction)
**Hayes 2014** [28]	**INTERVENTION COSTS:** $1309 per child (over 2 years); **ICER:** $2697 per unit BMI avoided and $376 per 0.1 reduction in BMI z-score (reduced travel time)
**Gortmaker 2015 (CHOICE)** [25]	**INTERVENTION COSTS in the 1**^**st**^** year (millions):** $51 for SSB, $1.1 for TV AD, $4.8 ECE, $71 for PE **ICER:** $1.16 per BMI unit change for TV AD, $3.16 for SSB, $ 57.80 for ECE and $401 for the Active PE intervention for the first 2 years. Substantial variations were in 10-year health outcomes (net cost saved per $ spent: $55 for SSB, $38 for TV AD, $6 for ECE)
**Gortmaker 2015 (Obesity & Diet)** [32]	**INTERVENTION COSTS per year ($ millions)/ ICER-per unit of BMI reduced ($)/Ten-year costs saved for $ spent:** Sugar-sweetened beverage excise tax: 47.6/2.49/30.78 Restaurant menu calorie labeling: 95.5/13.09/5.9 Elimination of the tax for advertising unhealthy food: 0.82/0.66/32.53 Nutrition standards for school meals: 1,112/53/0.42 Nutrition standards for food and beverages in schools: 22.3/6.1/4.56 Improved early care, education policies and practices: 76.0/613/0.04 Increased access to adolescent bariatric surgery: 30.3/1,61/not applicable
**Conesa 2018** [29]	**INTERVENTION COSTS:** 15.64 € per child or 5.21 €/child/year; 2.4 €/child/year to reduce the obesity prevalence in boys by 2% **ICER:** 968.66 € to avoid one case of obesity in boys (1.20 €/boy); 3.56 €/child to reduce the obesity prevalence by 1% in boys; 47.39 € for a decrease of one BMI unit per boy; 65.17 € for a decrease of one BMI z-score unit per boy
**Wyatt 2018** [27]	**INTERVENTION COSTS:** £144,749, with a mean estimated cost per child of £214; **ICER:** poor outcome, no evidence of positive differences in mean BMI SDS and in preventing overweight or obesity at 24-month follow-up
**Canaway 2019** [24]	**INTERVENTION COSTS:** £155.53 per child **ICER:** £26,815 per QALY < £30,000 willingness to pay threshold, with the probability of the intervention being cost-effective of only 52%
**Ananthap. 2019** [30]	**INTERVENTION COSTS:** AUD426M over 3 years **ICER:** AUD8155 per HALY gained (less than willingness to pay threshold for Australia of AUD 50,000 per HALY gained)
**Joo Tan 2020** [26]	**INTERVENTION COSTS** $184/child ($601/child for combination intervention) **ICER:** $18,125 per QALY gained and $1,022 per unit BMI avoided at age 5 years and $558 per unit BMI avoided at age 15 years

### Quality of studies

We used the Drummond Checklist to test quality assessment of the studies included in the systematic review (Table 3). [21, 22] We did not use the checklist for the review by Ş. Erdöl et al*.*, because of the presence of many studies in a single work. [23] All the studies are considered of low-medium quality. Five of the seven items are adherent to study design. Hayes et al. and J. Ananthapavan et al. did not state clearly the rationale for choosing the alternative programs. The presence of a justification for the form of economic evaluation is not clear in relation to the questions addressed in seven studies, except in the two studies by Gortmaker et al. Regarding data collection, eight items are totally adherent. Half of the studies gave details about the method of synthesis and meta-analysis of estimates. Only Wyatt et al. provided details about the subjects from whom evaluations were obtained, while only three studies reported quantities of resources separately from their unit costs. The majority of studies described methods for the estimation of quantities and unit costs, lastly only one gave some details of currency of price adjustments for inflation. Eight of them show information of the model used. There is a lack in the analysis and interpretation of results in many studies. In particular, nine of them are different in the following sections: the discount and the choice of rate, explanation if costs or benefits are not discounted, details of statistical tests and confidence intervals for stochastic data, the approach to sensitivity analysis, the choice of variables for sensitivity analysis, the ranges over which the variables are varied, comparing relevant alternatives, incremental analysis and major outcomes presented in a disaggregated as well as aggregated form.

**Table 3:** The Drummond checklist and the quality assessment of the studies included in the systematic review

**Study Design**	Kesztyu’s 2011 [31]	Hayes 2014 [28]	Gortmaker 2015 (CHOICE) [25]	Gortmaker 2015 (Obesity & Diet) [32]	Conesa 2018 [29]	Wyatt 2018 [27]	Canaway 2019 [24]	Ananthap. 2019 [30]	Joo Tan 2020 [26]
(I) The research question is stated	Y	Y	Y	Y	Y	Y	Y	Y	Y
(2) The economic importance of the research question is stated	Y	Y	Y	Y	Y	Y	Y	Y	Y
(3) The viewpoint(s) of the analysis are clearly stated and justified	Y	Y	Y	Y	Y	Y	Y	Y	Y
(4) The rationale for choosing the alternative programs or interventions compared is stated	Y	N	Y	Y	Y	Y	Y	NC	Y
(5) The alternatives being compared are clearly described	Y	Y	Y	Y	Y	Y	Y	Y	Y
(6) The form of economic evaluation used is stated	Y	Y	Y	Y	Y	Y	Y	Y	Y
(7) The choice of form of economic evaluation is justified in relation to the questions addressed	NC	NC	Y	Y	NC	NC	NC	NC	NC
**Data collection**									
(8) The source(s) of effectiveness estimates used are stated	Y	Y	Y	Y	Y	Y	Y	Y	Y
(9) Details of the design and results of effectiveness study are given (if based on a single study)	Y	Y	Y	Y	Y	Y	Y	Y	Y
(10) Details of the method of synthesis or meta-analysis of estimates are given (overview)	N	N	Y	Y	N	Y	N	Y	N
(11) The primary outcome measure(s) for the economic evaluation are clearly stated	Y	Y	Y	Y	Y	Y	Y	Y	Y
(12) Methods to value health states and other benefits are stated	Y	Y	Y	Y	Y	Y	Y	Y	Y
(13) Details of the subjects from whom valuations were obtained are given	NC	NC	NC	NC	NC	Y	NC	N	N
(14) Productivity changes (if included) are reported separately	N	N	N	N	N	N	N	N	N
(15) The relevance of productivity changes to the study question is discussed	N	N	N	N	N	N	N		N
(16) Quantities of resources are reported separately from their unit costs	Y	N	N	N	NC	N	Y	NC	Y
(17) Methods for the estimation of quantities and unit costs are described	Y	Y	NC	Y	Y	Y	Y	Y	Y
(18) Currency and price data are recorded	Y	Y	Y	Y	Y	Y	Y	Y	Y
19) Details of currency of price adjustments for inflation or currency conversion are given	N	N	N	N	N	N	N	N	Y
(20) Details of any model used are given	Y	Y	Y	Y	Y	Y	Y	NC	Y
(21) The choice of model used and the key parameters on which it is based are justified	N	N	N	N	N	N	N	N	N
**Analysis and interpretation of results**									
(22) Time horizon of costs and benefits is stated	Y	Y	Y	Y	Y	Y	Y	Y	Y
(23) The discount rate(s) is stated	N	Y		Y	N	N	Y	Y	Y
(24) The choice of rate(s) is justified	N		N	N	N	N	N	N	N
(25) An explanation is given if costs or benefits are not discounted	Y	NC	NC	NC	N	N	N	N	N
(26) Details of statistical tests and confidence intervals are given for stochastic data	Y	Y	Y	NC	Y	Y	Y	Y	Y
(27) The approach to sensitivity analysis is given	Y	Y	NC	Y	Y	Y	Y	Y	Y
(28) The choice of variables for sensitivity analysis is justified	N	N	N	N	N	Y	N	N	N
(29) The ranges over which the variables are varied are stated	Y	Y	Y	NC	Y	Y	Y	Y	Y
(30) Relevant alternatives are compared	N	N	Y	Y	Y	Y	Y	N	Y
(31) Incremental analysis is reported	Y	Y	NC	NC	Y	Y	Y	Y	Y
(32) Major outcomes are presented in a disaggregated as well as aggregated form	Y	N	N	N	N	NC	NC	Y	Y
(33) The answer to the study question is given	Y	Y	Y	Y	Y	Y	Y	Y	Y
(34) Conclusions follow from the data reported	Y	Y	Y	Y	Y	Y	Y	Y	Y
(35) Conclusions are accompanied by the appropriate caveats	Y	Y	Y	Y	Y	Y	Y	Y	Y

*Y* YES, *N* NOT, *NC* NOT CLEAR

## Discussion

Our review examined the cost-effectiveness analysis of primary prevention programs on overweight and obesity in children. The studies consider RCTs, overviews and systematic reviews. The heterogeneity of the studies in demographics, type of preventing programs, health and economic outcomes and time of follow-up is remarkable and did not allow specific comparisons. The preventing programs are based on nutrition and physical activity intervention mainly, while only one introduced the economic effect of the sleep. The main health outcomes evaluated are BMI changes and QALYs, a parameter of cost-utility analysis that evaluates the increase in average life expectancy corrected for the quality of the same. For all the studies, ICER is calculated like index of economic analysis and it is compared to national standard programs to evaluate if there is an economic advantage to apply the new intervention in childhood. We can conclude that the majority of the studies show cost-effective results acting on childhood lifestyle. The 70% of the works considered have positive economic results, so primary interventions could be applied to population with benefits. Only two studies considered primary prevention programs in preschool-aged children. In particular, the study by Hayes et al. shows moderately priced intervention, with an increased cost-effectiveness with reduction of travel-time for home visits, so it is not clear if there would be an advantage to adopt this prevention program, [28] while the study included in the overview paper by S. L. Gortmaker et al. considered changes in beverage, physical activity and screen time regulation of 3,69 million preschool children, for about two years. Probably this study will be cost-save by 2025, with net healthcare cost savings of $51.6 million [32, 33].

The other works examined intervention programs in school-aged children, with some limitations. With regards to the work of Gortmaker et al., only for three interventions the cost-effectiveness analysis resulted in a positive range: the sugar-sweetened beverage excise tax, eliminating the tax subsidy for advertising unhealthy food and setting nutrition standards for food and beverages sold in schools. [30] Instead, the program analyzed by Conesa et al. resulted cost-effective only for boys, limiting the significance of the intervention. [29] In light of this result, it could be evaluated if the differentiation of future prevention programs for male and females could be a significant intervention to improve cost-effectiveness. To date, there aren’t studies that show similar results.

Instead, the economic analysis by Canaway et al. on the WAVES program does not show clear results. [22] It is not certain that this intervention could be considered cost-effective, so we need other studies to highlight this aspect.

There is only one study with negative results, providing no significant differences in BMI SDS and WC SDS. [27] Thus, this school-based primary prevention program does not cause positive consequences if applied to population. In fact, very often prevention programs show their positive effects over a very long period of time; so the fact that these studies analyze the result over a period of a few years could be a limitation in the interpretation of the data result.

As for studies that focused on the cost-effectiveness over a long time, J. Ananthapavan et al. found positive economic effect adopting a community and school-based program, in particular with cost saving over a 29-year time horizon [30].

Lastly, there is a study that considered a new aspect in the prevention programs; indeed E. J. Tan et al. highlighted the importance of a sleep intervention for children, with health and economic consequences [26].

There is much evidence that insufficient sleep is a strong risk factor for overweight/obesity in children and adolescence [34, 35]. Children that sleep for a shorter duration have twice the odds of obesity compared with the control group. The cost-effectiveness analysis by Tan et al. proved that only sleep intervention is effective to prevent overweight, compared to combination group. [26] From our results it’s clear that both community and school-based programs have positive effects on health outcomes and cost-saving, so it could be necessary acting on both sides.

Indeed, none of the interventions would be sufficient alone to reverse the obesity epidemic. Policy makers need to develop a multifaceted prevention strategy that reaches individuals across the life course. Although there is much evidence of the efficacy of primary prevention on the risk of childhood obesity, data on economic evaluations are lacking and resources for policy makers and communities have limited resources to achieve the reduction in obesity.

Since the energy gap that drives excess weight gain is small, and adult obesity is difficult to reverse, interventions early on in life have the best chance of having an important impact on long-term obesity prevalence and related health care costs [32]. However, early intervention will not be sufficient if children living in a society that promote excess weight gain. Family history of obesity are important risk factors for precocious obesity onset in childhood and are related to the severity of.

obesity [36], this condition shows the importance of family interventions preventing childhood obesity. This problem is better showed in the HELENA study: eating habits of European adolescent are not healthy. In particular, they consume half the daily recommended quantities of fruit and vegetables and less than 2/3 of milk and dairy products, greater consumption of meat, fats and sweets, while vitamin D and folic acid deficiency is common [37]. The percentage of breakfast skippers vs consumers is 50% vs 50% [38]. Lastly, physical education doesn’t meet CDC recommended levels and only 30% of children aged 6–11 years in the USA reach these levels [39].

In conclusion, the investment in childhood obesity prevention is important to have the maximum benefit in adulthood for the prevention of chronic disease. In fact, a high BMI in childhood persists in adulthood, so child weight status can be a predictor of adult weight status in a policy context [40].

## Conclusions

The majority of the studies reveals a positive effect on children’s well-being, so the interventions are considered effective in the reduction of BMI, WC and other anthropometric measures. Most of the interventions proved to be low-cost; therefore, policy-makers should promote and undertake them to save economic resources, as well as improve general health. We need additional studies analyzing primary interventions that use homologous health outcomes and that reduce differences between population and cost-effectiveness analysis in order to evaluate better prevention programs. We propose cardiovascular outcomes in obese children (e.g. high blood pressure, initial changes in heart structure and function) for cost-effectiveness analysis in future studies. Moreover, it is important to examine the difference between the efficacy of lifestyle change between preschool and school children. In this way, we can have an instrument available to reduce health costs both in short and in long-term outcomes.

## Data Availability

Data sharing is not applicable to this article as no datasets were generated or analyzed during the current study.

## Abbreviations

CEA: Cost-effectiveness analysis
BMI: Body mass index
PA: Physical activity
WC: Waist circumference
LVM: Left ventricular mass
LVH: Left ventricular hypertrophy
DALYs: Disability-adjusted life years
QALYs: Quality-adjusted life years
ICER: Incremental cost-effectiveness ratio
RCTs: Randomized clinical trials
HALYs: Health-adjusted life years
WHtR: Waist-to-height ratio
URMEL-ICE: Ulm Research on Metabolism, Exercise and Lifestyle Intervention in Children
MWTP: Maximum willingness to pay
HB: Healthy Beginnings
CHOICES: Childhood Obesity Intervention Cost-Effectiveness Study
SSB: Sugar-sweetened beverages
TV AD: TV advertisements
PE: Physical education
ECE: Early child education
EdAI: Educació en Alimentació
SDS: Standard deviation score
WAVES: The West Midlands ActiVe lifestyle and healthy Eating in School children
CBIs: Community-based obesity prevention interventions
POI: Prevention of Overweight in Infancy
FAB: Food, activity, and breastfeeding advice
